# A scoping review on the factors associated with the lost to follow-up (LTFU) amongst patients with chronic disease in ambulatory care of high-income countries (HIC)

**DOI:** 10.1186/s12913-023-09863-0

**Published:** 2023-08-22

**Authors:** Ching Yi Michelle Tong, Rui Ying Victoria Koh, Eng Sing Lee

**Affiliations:** 1 National Healthcare Group Polyclinics, Singapore, Singapore; 2 MOH Office for Healthcare Transformation, Singapore, Singapore; 3 Lee Kong Chian School of Medicine, Nanyang Technological University, Singapore, Singapore

**Keywords:** Lost to follow-up, Chronic disease, Mental health, Physical health, Ambulatory care, High-income countries

## Abstract

**Background:**

Despite the importance of long term follow-up care for patients with chronic disease, many patients fail to adhere to their follow-ups, which increase their risk of further health complications. Therefore, the purpose of this scoping review was to find out the factors associated with lost to follow-up (LTFU) amongst patients with chronic disease in the ambulatory care setting of high-income countries (HICs) to gain insights for better quality of care. Understanding the definition of LTFU is imperative in informing patients, health professionals and researchers for clinical and research purposes. This review also provided an overview of the terms and definitions used to describe LTFU.

**Methods:**

The following databases: CINAHL, EMBASE, Medline, PsycINFO and Web of Science were searched for studies investigating the factors associated to LTFU from the date of inception until 07 January 2022.

**Results:**

Five thousand one hundred and seven records were obtained across the databases and 3,416 articles were screened after removing the duplicates. 25 articles met the inclusion criteria, of which 17 were cohort studies, five were cross-sectional studies and three were case-control studies. A total of 32 factors were found to be associated with LTFU and they were categorised into patient factors, clinical factors and healthcare provider factors.

**Conclusion:**

Overall, the factors associated with LTFU were generally inconsistent across studies. However, some factors such as financial factors (i.e., no insurance coverage) and low accessibility of care were consistently associated with LTFU for both mental and physical chronic conditions. The operational definitions of LTFU also varied greatly across studies. Given the mixed findings, future research using qualitative aproaches would be pivotal in understanding LTFU for specific chronic diseases and the development of targeted interventions. Additionally, there is a need to standardise the operational definition of LTFU for research as well as clinical practice purposes.

**Supplementary Information:**

The online version contains supplementary material available at 10.1186/s12913-023-09863-0.

## Introduction

Chronic diseases impose a significant burden on individuals, households, health systems and global economies through increased disability [1], higher health care expenses [2, 3] and lost productivity [4]. As there is no universal consensus on the definition of chronic disease [5], we adopted the definition described by Tyagi et al. which states that a chronic disease is defined as a medical condition lasting for six months or more; be recurrent or have a persistent course; impacts the patient physically, or psychologically or reduces lifespan; and requires long-term follow-up [6]. Patients with chronic disease require continual follow-up with a care team. However, adhering to long-term follow-up is challenging as being lost to follow-up (LTFU) is a prevalent issue among patients with chronic disease [7, 8]. Patients who are LTFU face poorer disease control [9, 10], higher risk of hospitalisation [11] and mortality [12, 13]. Given that a significant proportion of individuals in high-income countries (HIC) are living to older ages [14, 15] and aging is a risk factor of chronic disease, LTFU among chronically ill individuals is a public health issue that calls for attention in HICs.

It is crucial to identify the factors associated with LTFU to improve the quality of care and support for patients with chronic disease. To date, based on the authors’ knowledge, there are limited reviews investigating the factors associated with LTFU among patients with chronic disease in HICs. Previous systematic reviews mainly investigated the factors associated with missed appointments, focusing on a specific chronic disease such as diabetes mellitus (e.g., Brewster et al., 2020; Lee et al., 2019; Sun et al., 2021 [16–18]). While these reviews offer useful insights, they are limited to a small range of chronic physical diseases [16–19]. With the growing concern of chronic mental diseases such as Alzheimer’s disease contributing to mortality in HICs [20], studying the factors associated with LTFU for a wider scope of chronic diseases is warranted. Moreover, existing reviews included studies from countries of varying income levels [16–18], thus the findings may not be specific to HICs. Although the health systems in HICs and low- and middle-income countries (LMICs) both strive to provide quality and continuous care while reducing costs, HICs and LMICs operate with very different healthcare structure and approaches [21]. For instance, LMICs prioritises the development of specific groups of services (e.g., family planning), communicable disease programmes and increasing access to basic healthcare needs [21]. Whereas HICs focuses on providing quality care for patients with multiple chronic diseases, improving patient experience and promoting patient self-management [21]. The difference in focus, availability, and quality of care services delivered in LMICs and HICs may influence the factors associated with LTFU in the respective countries of varying income levels. Therefore, in order to gain a better understanding of specific factors associated with LTFU in HICs, it is important to investigate LTFU in HICs and LMICs independently.

Furthermore, while the concept of LTFU has been employed widely in clinical settings to identify patients who may have disengaged from care (e.g. Chi et al., 2011) [22], the frequent use of this concept in other fields and contexts creates ambiguity to its meaning in the field of healthcare [23]. The current literature also lacks a standardised measure or definition of LTFU for healthcare appointments [23, 24]. Therefore, gaining clarity and understanding the definition of LTFU within the healthcare setting serves a pivotal function from both a research and clinical perspective [24], benefiting patients, healthcare providers and researchers. It would also be essential to obtain an overview of the terms and definitions used to describe LTFU prior to establishing a uniform definition of LTFU.

In order to gain a broad understanding of the factors associated with LTFU among patients with various chronic diseases in HICs, a scoping review was conducted. The main aim of this scoping review was to systematically identify the factors associated with LTFU amongst patients with chronic disease in the ambulatory care setting of HICs. The secondary aim was to provide an overview of the terms and definitions used to describe LTFU in the included studies.

## Methods

This scoping review adopted Arksey and O’Malley’s methodological framework [25] and the reporting guidelines suggested in the PRISMA Extension for Scoping Reviews [26]. A review protocol was registered and published in Open Science Framework under the following registration https://doi.org/10.17605/OSF.IO/45J2Q.

### Defining the research question

This review was guided by the primary question: “What are the factors associated with LTFU amongst patients with at least one chronic disease in an ambulatory care setting within HIC?”.

### Identifying relevant studies

A literature search was conducted in the CINAHL, EMBASE, Medline (Ovid), PsycINFO (Ovid) and Web of Science databases using the medical subject headings and keywords found in the supplementary file (see Appendix A for the search strategy). All chronic diseases that fulfilled the criteria described by Tyagi et al. [6] were included in this review. The search was limited to studies published in English from database inception to 07 January 2022. The search strategies were drafted in consultation with a health sciences librarian and refined through team discussions amongst the authors.

### Study selection

Studies were included if they fulfilled the following criteria: (1) study’s objective involved assessing the factors associated with LTFU in routine care; (2) included adult patients with at least one chronic disease in the ambulatory care setting; (3) carried out in HICs according to the classification of the World Bank [27]; (4) were peer-reviewed observational studies, either quantitative or mixed methods. Studies were excluded if they investigated LTFU during transition from paediatric to adult care or LTFU when a specific programme or modality (i.e., not usual standard clinical care) of treatment was used. Studies that did not adjust for potential confounding factors were excluded. Reviews, meta-analyses, case reports, case studies as well as interventional studies were also excluded from the review. In light of the absence of a standardised definition of LTFU for healthcare appointments [23], this review considered LTFU as a significant gap in follow-up appointments when patients miss their scheduled appointments and return with a considerable delay or never return [28].

Two reviewers (TCYM and VKRY) independently screened the titles and abstracts then reviewed the full texts of all potentially relevant articles based on the inclusion and exclusion criteria. Disagreements on article selection were resolved by consensus and discussion with another reviewer (LES).

### Data charting

A data-extraction form was developed to extract data including author(s), year of publication, study location, study design, care setting, disease studied, category of disease (mental health, physical health, unspecified), study objective(s), age, factors associated with LTFU, covariates, LTFU definition, LTFU duration and terminology and type of statistical analysis.

When the research team required further clarification regarding specific articles, the clarification was sought from corresponding authors via email. If the authors did not respond, uncertainties were discussed among the reviewers until a consensus was reached.

### Collating, summarising, and reporting results

A narrative synthesis approach was adopted in summarising the findings and the authors inductively classified the results into the following categories: patient, clinical and healthcare provider factors. Some studies which fulfilled the overall inclusion criteria also included a qualitative component (i.e., anecdotal reasons provided by patients for their LTFU). Since these qualitative reasons did not undergo statistical analysis, they were presented separately from ‘factors’ in our review. The terminology and definitions of LTFU in the included studies were also captured and summarised.

## Results

### PRISMA 

**Fig. 1:**
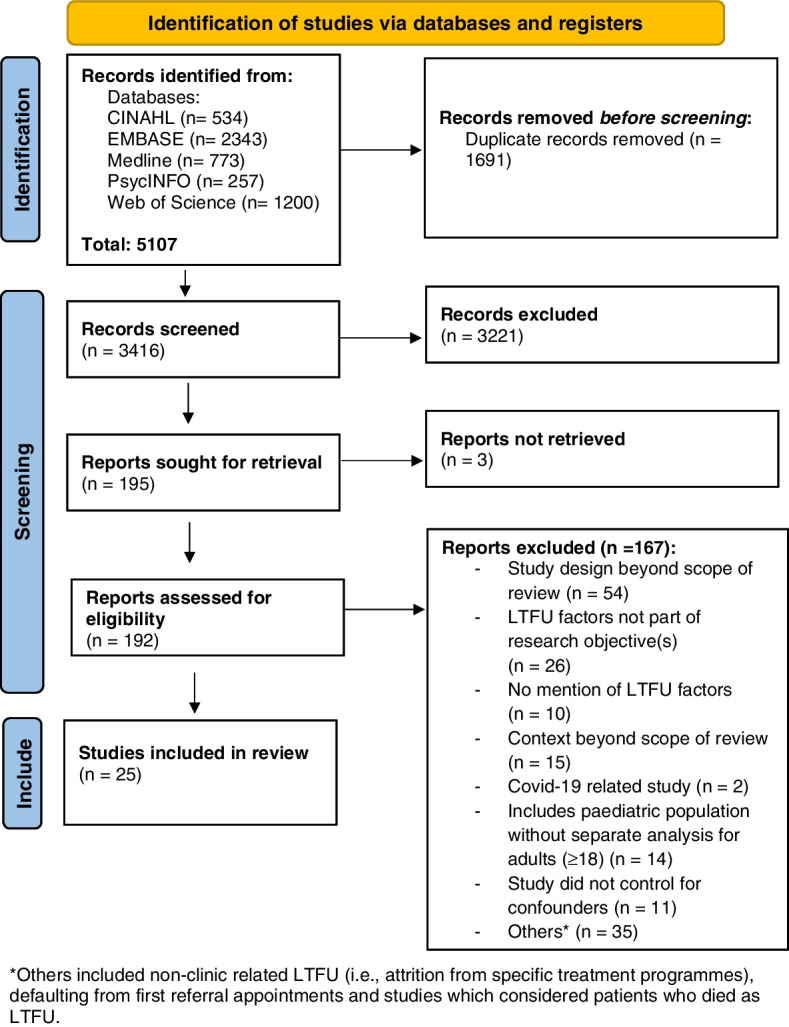
PRISMA flowchart of the study selection process

A total of 5,107 records were obtained from the searches, comprising of 534 CINAHL, 2,343 EMBASE, 773 Medline, 257 PsycINFO and 1,200 Web of Science articles. After the removal of duplicates, 3,416 records were screened and 195 were selected for full-text assessment after reviewing their titles and abstracts. Ultimately, 25 articles were included in this review as presented in the PRISMA flowchart [29] (Fig. 1).

### Characteristics of the included studies

Among the 25 articles, 17 were cohort studies [30–46], five were cross-sectional studies [47–51] and three were case-control studies [52–54]. The study characteristics such as the country, study design, disease, care setting, sample size etc. of the included articles are presented in Table 1. The studies were conducted over a range of HICs. One study was carried out across several countries, namely Argentina, Belgium, France, Germany, Israel, Italy, Japan, Netherlands, New Zealand, Northern Ireland, Poland, Portugal, Spain, and USA [48]. The remaining studies were conducted in single countries. Eleven of them were carried out in the USA [30, 32, 33, 35, 37, 39, 42, 43, 45–47], four in Japan [36, 38, 40, 51], two in Hong Kong (Special Administrative Region), China [50, 52], two in South Korea [41, 53], and one each in Austria [31], Canada [44], Israel [49], New Zealand [54] and Spain [34].

**Table 1:** Characteristics of included studies

Study/Country,Study Design	Care setting & Disease	n	Patients LTFU/ (%)	Age (years) Mean (SD) / Median (IQR) / Range				Lost to follow-up (LTFU) Definition	Time frame of LTFU definition (months) (How time frame was derived?)	How was LTFU identified?
**Chronic Mental Disease**										
Berghofer et al. (2002) [31]/ Austria, Cohort	Community mental health centres Patients with mental health condition except those with dementia or mental retardation	111	57 (51.4%)	**Non-LTFU: LTFU**: **Overall:**	41.3 (13.4) 35.9 (12.2) 38.5 (13.0)	DNS DNS DNS	DNS DNS DNS	Defined dropouts as patients who from the clinical point of view need psychiatric treatment, but who discontinue treatment on their own initiative by failing to attend arranged visits at the Community Mental Health Centres during the 4-month study period	4 (Prior analysis indicated stabilisation of service use after 4 months)	Self-reported
Bowersox et al. (2013) [32]/ USA, Cohort	Outpatient Veterans Health Administration care Mood disorder; Psychosis; Substance dependence disorder; Anxiety disorder; Axis II disorder	233	202 (86.7%)	**Non-LTFU:** **LTFU**: **Overall:**	DNS DNS 49.89 (8.28)	DNS DNS DNS	DNS DNS DNS	Dropped out of care based on a period of 5 weeks or greater without contact with their treatment staff	1 15 (5 weeks) (Validated standard by the Health Plan Employer Data Information set)	Electronic medical records
Boyd et al. (2022) [33]/ USA, Cohort	Memory and Aging Center Dementia	746	314 (42.1%)	**Non-LTFU:** **LTFU**: **Overall:**	DNS DNS DNS	DNS DNS DNS	DNS DNS DNS	Follow‐up status was determined from the documented follow-up plan at the final in‐person clinic visit. Participants who did not return to clinic 3 or more months after their recommended return date were classified as LTFU	≥ 3 (DNS)	Documented follow-up records at final in-person clinic visit
Ezquiaga et al. (2014) [34]/ Spain, Cohort	Mental Health Centers and Psychiatric outpatient clinic (General hospital) Bipolar disorder (I and II)	285	115 (40.4%)	**Non-LTFU:** **LTFU**: **Overall:**	DNS DNS 47.8 (14.1)	DNS DNS DNS	DNS DNS DNS	Dropout included patients who failed to attend appointments for more than 6 months	≥ 6 (DNS)	Documented follow-up records
Fernandez et al. (2021) [48]/ Multiple countries^a^, Cross-sectional	Outpatient mental healthcare DSM-IV mental disorders (mood disorders; anxiety disorder; externalising disorders; substance disorders)	3787	1108 (29.3%)	**Non-LTFU:** **LTFU**: **Overall:**	DNS DNS DNS	DNS DNS DNS	DNS DNS 18 to 100	Respondents who reported quitting before the provider(s) wanted them to stop were classified as having dropped out from that treatment sector	DNS	Self-reported
Hishikawa et al. (2017) [36]/ Japan, Cohort	Memory clinics (affiliated to UH and LH) Dementia	Total: 988 UH: 383 LH: 605	Total: 394 (39.9%) UH: 207 (54.0%) LH: 187 (31.0%)	**Non-LTFU:** **LTFU**: **Overall:**	78.8 (7.2) 79.8 (8.7) DNS	DNS DNS DNS	DNS DNS DNS	Medical records were reviewed for 5 years from January 2011 to December 2015 to identify patients who discontinued (No formal definition of LTFU was provided except time range of reviewed medical records.)	DNS	Medical records
Lerner et al. (2012) [49]/ Israel, Cross-sectional	General medical health clinic and specialized mental health clinics Panic disorder; Generalized anxiety disorder; Agoraphobia; Post-traumatic stress disorder; Mood disorder	275	66 (24.0%)	**Non-LTFU:** **LTFU**: **Overall:**	DNS DNS DNS	DNS DNS DNS	DNS DNS ≥ 21	Respondents who reported quitting before the healthcare provider wanted them to stop were classified as having dropped out	DNS	Self-reported
Minamisawa et al. (2016) [40]/ Japan, Cohort	University outpatient clinic Mood disorder; Anxiety disorder	532	190 (35.7%) Mood = 59 Anxiety = 131	**Non-LTFU:** **LTFU**: **Overall:**	DNS DNS DNS	DNS DNS DNS	DNS DNS ≥ 18	Patients who had failed to return to the hospital for the next appointment without their psychiatrist’s consent, and did not come back to continue treatment for 90 days based on the definition proposed by Tansella et al. (1995)	3 (Proposed by prior research studies)	Medical records
Moon et al. (2012) [41]/ South Korea, Cohort	Mood disorder clinic Bipolar disorder (I and II)	275	138 (50.2%)	**Non-LTFU:** **LTFU**: **Overall:**	DNS DNS 39.4 (12.4)	DNS DNS DNS	DNS DNS DNS	Dropout was defined as a patient stopping treatment for longer than one month without receiving other treatment despite a psychiatrist's recommendation of treatment maintenance by Miller et al. (2009)	> 1 (Proposed by prior research studies)	Medical records
Simon et al. (2010) [42]/ USA, Cohort	Mental health clinic Depression	238	Dropped out before second visit, *n* = 103 (43.3%) Dropped out before first visit, *n* = 53 (22.3%)	**Non-LTFU:** **LTFU**: **Overall:**	DNS DNS DNS	DNS DNS DNS	DNS DNS ≥ 18	Dropout before first visit after requesting psychotherapy for treatment of depression or dropout before second visit over the study period of 90 days	3 (DNS)	Insurance claims data for psychotherapy visits
Sirey et al. (2001) [43]/ USA, Cohort	Psychiatric outpatient clinic Major depressive disorder	92	16 (17.4%)	**Non-LTFU:** **LTFU**: **Overall:**	DNS DNS DNS	DNS DNS DNS	DNS DNS ≥ 18	Our treatment outcome measure was a dichotomous variable classifying patients as either “continued in treatment” or “discontinued treatment” (and did not seek treatment elsewhere) during the 3-month follow-up period	3 (DNS)	Self-reported and clinical chart records
**Chronic Physical Disease**										
Ballard et al. (1988) [30]/ USA, Cohort	Hypertensive clinic Hypertension	641	135 (21.1%)	**Non-LTFU:** **LTFU**: **Overall:**	DNS DNS DNS	DNS DNS DNS	DNS DNS DNS	HyperteDNSives who did not have a clinic visit with a blood pressure recorded in the year prior to their record review data were coDNSidered to be lost to follow-up	DNS	Medical records
Buys et al. (2019) [47]/ USA, Cross-sectional	Outpatient clinic Diabetes	139	62 (44.6%)	**Non-LTFU:** **LTFU**: **Overall:**	46 (11.4) 44 (10.4) 44 (12.2)	DNS DNS DNS	DNS DNS DNS	Lost to follow-up is defined as patients who attended at least 1 visit and had a follow-up scheduled but failed to attend their most recently scheduled visit at least 30 days prior; this included only patients who did not call to cancel their appointment	≥ 1 (DNS)	Electronic medical record
Chow et al. (2011) [52]/ Hong Kong SAR, China, Case-control	Predialysis low clearance clinic Chronic kidney disease (stage IV or V)	112	36 (32.1%)	**Non-LTFU:** **LTFU**: **Overall:**	63.3 (14.5) 54.7 (16.1) DNS	DNS DNS DNS	DNS DNS DNS	Patients who did not attend their scheduled clinic appointments without administrative or medical reasons (hospitalization or death) were defined as cases. Patients who missed the clinic appointment, but subsequently reappeared because of disease complications, were included as cases	DNS	Attendance records
Gao et al. (2019) [35]/ USA, Cohort	Hospital and outpatient retina practice Macular edema secondary to retinal vein occlusion	3400	863 (25.4%)	**Non-LTFU:** **LTFU**: **Overall:**	DNS DNS 75.3 (13.2)	DNS DNS DNS	DNS DNS DNS	LTFU was defined as a subsequent visit occurring more than 12 months after an intravitreal injection or no further visits after the last intravitreal injection with this last injection occurring at least 12 months prior and no records of death before the end of the study period. (Window for observation: Jan 1, 2016 to Jan 1, 2017)	12 (DNS)	Database records
Khanh et al. (2020) [37]/ USA, Cohort	Outpatient vascular clinic Vascular disease (endovascular aneurysm repair; carotid endarterectomy; infrainguinal bypass; peripheral vascular intervention)	440	326 (74.1%)	**Non-LTFU:** **LTFU**: **Overall:**	DNS DNS DNS	DNS DNS 67 (62–75)	DNS DNS DNS	Primary end point was LTFU at 1 month, which was defined as lack of an inperson vascular clinic visit within 30 days of discharge from the index procedure. Secondary end points were LTFU at 1 year, defined as lack of an in-person vascular clinic visit within 9 and 22 months after discharge from index procedure)	Primary: 1 month post-discharge (Standard practice in given division) Secondary: 9–22 months post-discharge (NS)	Electronic medical record
Kim et al. (2021) [53]/ South Korea, Case-control	University hospital Diabetic macular edema	182	60 (33.0%)	**Non-LTFU:** **LTFU**: **Overall:**	56.73 (12.28) 57.80 (12.28) DNS	DNS DNS DNS	DNS DNS DNS	Patients who had a history of LTFU for more than 1 year after the last visit were assigned to the LTFU group	12 (DNS)	Database records
Masuda et al. (2006) [38]/ Japan, Cohort	Diabetes clinic Type 2 Diabetes Mellitus	160	68 (42.5%)	**Non-LTFU:** **LTFU**: **Overall:**	58.66 (11.11) 53.74 (13.44) DNS	DNS DNS DNS	DNS DNS DNS	Patients who had not visited the diabetes clinic for at least 12 months since their last visit from 1 August 2000 to 31 August 2001, including visits to other clinics, or hospitals, or dropout from diabetic care	12 (DNS)	Database records
Mathieu et al. (2014) [39]/ USA, Cohort	Academic medical centre Gestational Diabetes Mellitus	373	186 (49.9%)	**Non-LTFU:** **LTFU**: **Overall:**	DNS DNS 31.0 (4.8)	DNS DNS DNS	DNS DNS DNS	Failure to attend follow-up is defined as a not returning to the endocrinology clinic within 4 months of delivery for the purposes of GDM follow-up	4 (Clinically recommended standard)	Database records
Shiu et al. (2019) [50]/ Hong Kong SAR, China, Cross-sectional	General outpatient clinic Diabetic retinopathy	400	120 (30.0%)	**Non-LTFU:** **LTFU**: **Overall:**	64.45 (10.98) 64.20 (10.60) DNS	DNS DNS DNS	DNS DNS DNS	Non-attenders were defined as subjects who did not attend any scheduled diabetic retinopathy screening appointments from 1st January 2013 to 31st December 2013 and attended the clinic again from 1st March 2014 to 30th April 2014 for diabetic retinopathy screening	12 (Clinically relevant interval for follow-up ranges from 6 to 24 months)	Database records
Simmons et al. (2007) [54]/ New Zealand, Cross-sectional	Primary care Type 2 Diabetes Mellitus	89	37 (41.6%)	**Non-LTFU:** **LTFU**: **Overall:**	56.0 (11.0) 55.0 (14.0) DNS	DNS DNS DNS	DNS DNS DNS	“Defaulters” were defined as those reporting no diabetes care from any health professionals in the previous 10 months (taken as it implies three missed quarterly appointments)	10 (Prior research interval)	Self-reported
Sonoda et al. (2020) [51]/ Japan, Cross-sectional	Outpatient clinic Type 2 Diabetes Mellitus	140	12 (8.6%)	**Non-LTFU:** **LTFU**: **Overall:**	50.6 (5.7) 45.1 (4.4) DNS	DNS DNS DNS	DNS DNS > 20	Outpatient visit status was obtained using a self-administered questionnaire. Partcipants who selected "I have dropped out of outpatient diabetes treatment visits" were classified as dropout group	DNS	Self-reported
Szadkowski et al. (2018) [44]/ Canada, Cohort	Tertiary care centre Human immunodeficiency viruses (HIV)	1591	395 (24.8%)	**Non-LTFU:** **LTFU**: **Overall:**	DNS DNS DNS	DNS DNS 42 (35 to 49)	DNS DNS DNS	Intervals between consecutive visits with an HIV specialist (inter-visit intervals) were classified as greater than or less than 12 months	> 12 (Clinically relevant interval)	Database records
Tsui et al. (2016) [45]/ USA, Cohort	Veterans Affairs Medical Center Diabetes (eye screening)	120	45 (37.5%)	**Non-LTFU:** **LTFU**: **Overall:**	DNS DNS 65 (DNS)	DNS DNS DNS	DNS DNS DNS	Lost to follow-up rates for eye care were defined as no eye clinic or teleretinal screening within 2 years of the index teleretinal screening visit	24 (DNS)	Database records
**Unspecified Chronic Disease**										
Yoon et al. (2020) [46]/ USA, Cohort	Primary care under Veterans Health Administration practices Veterans with at least one chronic condition identified through the Gagne comorbidity score	1522969	67703 (4.5%)	**Non-LTFU:** **LTFU**: **Overall:**	DNS DNS DNS	DNS DNS DNS	DNS DNS 20 to 63	Attrition from Veterans Health Administration primary care, was measured for patients who used Veterans Health Administration primary care during FY2015 and did not receive any Veterans Health Administration primary care visits during two subsequent years (FY2016-2017) based on prior research on attrition	24 (Prior research on attrition)	Database records

*DNS* Did not specify, *IQR* Interquartile range, *LH* Local hospital, *LTFU* lost to follow-up, *SD* Standard deviation, *UH* University hospital

^a^Multiple countries included: Argentina (not classified as High Income Country in the 2021 World Bank List); Belgium; France; Germany; Israel; Italy; Japan; Netherlands; New Zealand; Northern Ireland; Poland; Portugal; Spain; United States

The chronic diseases studied ranged from mental to physical health conditions such as depression to diabetes mellitus. For the purpose of this review, these studies were broadly classified into three categories: “Chronic mental disease”, “Chronic physical disease” and “Unspecified chronic disease” for articles that did not explicitly mention the type of chronic disease studied. Each category included 11 [31–34, 36, 40–43, 48, 49], 13 [30, 35, 37–39, 44, 45, 47, 50–54] and one [46] study respectively. For chronic mental disease studies, the diseases included: anxiety disorder [40, 49], bipolar disorder [34, 41], dementia [33, 36], depression [40, 42, 43]. For chronic physical disease studies, the diseases included: diabetes [38, 39, 47, 51, 54] or diabetes-related conditions [35, 45, 50, 53], human immunodeficiency virus (HIV) [44], hypertension [30], kidney disease [52] and vascular disease [37].

The mean age of patients varied across studies — ranging from 35.9 [31] to 79.8 [36] years old. Of the included studies, three were conducted among young adults [31, 39, 41], eight among middle-aged adults [32, 34, 38, 47, 51–54] and four among the elderly population [35, 36, 45, 50]. Mean age was not reported in the remaining 10 studies [30, 33, 37, 40, 42–44, 46, 48, 49]. The earliest article included in this review was published in 1988. There were more articles published over the recent years.

### Factors associated with LTFU

Statistically significant factors associated with LTFU among patients with chronic disease in the ambulatory care setting of HICs are summarised in Table 2. A detailed version with the directionality and covariates of each study is included in the supplementary material (Table A). Covariates, especially those of age [30–35, 38–44, 46–52, 54] and sex [31–33, 35, 38, 40–42, 44, 46–50, 53], were adjusted in most of the studies. Overall, 32 factors were found to be associated with LTFU. They can be broadly classified into patient, clinical and healthcare provider factors.

**Table 2:** Significant factors associated with LTFU

	**Chronic Mental Disease** (*n* = 11)	**Chronic Physical Disease** (*n* = 13)	**Any Chronic Disease** (*n* = 1)
**Patient Factors**			
**Demographics**			
1. Age	[32, 40]	[35, 44, 51, 52]	
2. Gender/Sex	[32, 33, 42]	[50, 53]	
3. Ethnicity/Race		[35, 44]	
4. Education level	[33, 40]	[39]	
5. Marital status	[40]		
6. Employment status	[31]		
7. Home care availability	[31]		
8. Living situation (alone/not alone)	[31]		
9. Distance between home and clinic	[33]	[35, 45]	
10. Health insurance	[48]	[35]	
11. Place of residence	[33]		
**Drug and tobacco use**			
12. Drug use		[44]	
13. Smoking status	[34]		
**Knowledge/beliefs/attitudes**			
14. Perceived satisfaction with life	[31]		
15. Perceived stigma	[32, 43]		
**Others**			
16. History of compliance	[32, 34, 41]		
17. Supervisor support at work		[51]	
**Clinical Factors**			
**Disease Factor**			
18. Any history of mental illness	[31, 41]		
19. Baseline health status		[30, 35, 39, 51]	
20. Cognitive function	[36]		
21. Current diagnosis	[31]		
22. Disease complications		[35, 50, 53, 54]	
23. Duration of disease	[41]	[44, 53]	
24. Postoperative complications		[37]	
25. Seasonality	[34]		
26. Severity of condition/disease	[31, 40, 42, 48]	[39, 44, 50]	
**Medication Factor**			
27. Specific medications		[38, 54]	
**Healthcare Provider Factors**			
28. Interaction with healthcare facility/provider	[32]	[30, 37, 44]	
29. Patient-physician sex concordance	[40]		
30. Physician's drug aggressiveness		[30]	
31. Quality of health provider services	[31]		[46]
32. Treatment setting	[49]		

#### Patient factors

A total of 17 patient factors from 17 studies [31–35, 39–45, 48, 50–53] were identified (Table 2). The studies explored various patient factors that potentially influenced the failure of patients to return for follow-up appointments at their respective health clinics. The results were summarised under the following categories: demographics, drug and tobacco use, knowledge/ beliefs/ attitudes, and others.

Several mental and physical disease studies reported that younger age was positively associated with LTFU [32, 40, 44, 52] and older patients were less prone to LTFU [35, 51]. However, majority of the studies did not find an association between age and LTFU [30, 31, 33, 34, 38, 39, 41–43, 46–50, 54]. Most studies also reported no association between sex and LTFU [31, 35, 38, 40, 41, 44, 46–49]. Of those that found an association, findings were mixed. Two reported that males were positively associated to LTFU [32, 53], while another study found a negative relationship between males and LTFU [42]. Similarly, females were reported to be either positively [33] or negatively [50] associated with LTFU. Many studies reported that race was not associated to LTFU [32, 33, 39, 46, 47, 54]. Only two studies found a significant association between race and LTFU. However, the findings were contradictory. One study reported that non-white race was positively associated to LTFU [35], whereas another study found that white race was positively associated to LTFU [44]. While three studies found that lower education level was positively associated to LTFU [33, 39, 40], other studies reported no association [31, 48, 50, 54]. Marital status was mainly investigated in chronic mental disease studies only [31, 40, 46, 48] and one study found that being divorced or widowed was positively associated with LTFU [40]. While most studies did not find an association between employment status or type and LTFU [40, 48, 54], one study found a positive association between unemployment and LTFU [31]. Living conditions such as home care availability and living alone were negatively associated with LTFU [31] and patients who stayed and passed away in long-term care facilities were positively associated with LTFU at their outpatient clinics [33]. Most studies [33, 35, 45] showed that further distance between home and clinic was positively associated with LTFU, except one study which did not find any relationship [46]. Patients without health insurance coverage were positively associated with LFTU [35, 48].

For illicit drug use, a chronic mental disease study showed no association with LTFU [34]. Mixed findings were reported for tobacco use. While a chronic physical disease study and chronic mental disease study found that smoking was positively associated with LTFU [34, 44], another chronic physical disease study reported no association between the two [54]. As for knowledge, beliefs and attitudes, LTFU patients were negatively associated with high perceived quality of life [31] and positively associated with high perceived stigma [32, 43]. Sirey et al. [43] reported that the positive association between greater perceived stigma and LTFU was present in older but not younger patients. Other patient factors associated with LTFU included history of compliance, whereby poorer history of compliance [34], previous history of dropout [41] and less frequent medication pick-up [32] were positively associated with LTFU. This was only investigated in chronic mental disease studies. Higher supervisor support at work was negatively associated with LTFU [51].

#### Clinical factors

Nine clinical factors categorised into disease and medication factors respectively were identified from 17 studies [30, 31, 34–42, 44, 48, 50, 51, 53, 54]. Several studies reported that baseline health status was associated with LTFU [30, 35, 39, 51]. More specifically, higher baseline Body Mass Index (BMI) [30, 39], and poor baseline visual acuity [35] was positively associated with LTFU, and the presence of metabolic syndrome at baseline was negatively associated with LTFU [51]. Other baseline health factors such as diastolic [30, 54] and systolic [54] blood pressure were not associated with LTFU. Two [31, 41] out of three [31, 40, 41] chronic mental disease studies found a relationship between mental illness history and LTFU. Receiving previous psychiatric treatment was positively associated with LTFU [31] and having past mental disorder diagnosis was negatively associated with LTFU [41]. A study also found an association between the cognitive functions and LTFU whereby worsening cognitive function was positively associated with LTFU at a memory clinic [36].

For the presence of disease complications, chronic physical disease studies showed varied association, with some studies reporting positive [50, 53], negative [35, 54] or no association [45] with LTFU. While some studies showed no association between the duration of chronic disease and LTFU [34, 50, 54], others found an association [41, 44, 53]. Generally, the findings were consistent such that a longer disease duration was negatively associated with LTFU [41] and a shorter duration was positively associated with LTFU [53]. However, for patients infected with HIV, longer inter-visit intervals (i.e., LTFU) was positively associated with longer duration of HIV [44]. Several chronic mental [31, 40, 42, 48] and chronic physical [39, 44, 50] disease studies also found an association between disease severity — assessed using various parameters relevant to the chronic disease (e.g., HbA1c level for diabetes, patient health questionnaire for depression, viral load copies for HIV etc.) — and LTFU. Some reported that higher disease severity was positively associated [39, 48, 50] with LTFU while others reported a negative association [40, 42]. A mental health study [31] found that current diagnosis type was associated with LTFU. Patients diagnosed with schizophrenia were less prone to LTFU compared to diagnosis of other mental health conditions. Seasonality was also positively associated with LTFU in another chronic mental disease study [34].

Under medication factors, specific medications such as insulin treatment and type of medications were associated with LTFU [38, 54]. However, this association was mainly found in chronic physical disease studies only.

#### Healthcare provider factors

A total of five factors related to healthcare providers from eight studies [30–32, 37, 40, 44, 46, 49] were identified and presented in Table 2. For both chronic mental [32] and chronic physical [30, 44] disease studies, interactions with healthcare facility or provider were associated with LTFU. Fewer short-term therapy attendance [32] and less intense contact with the medical care system [30] were positively associated with LTFU. LTFU is negatively associated with patient-physician sex concordance [40] and positively associated with physicians who were low in drug aggressiveness [30], treatment under general medical sector [49] and medical centre-based clinics [46]. Poor quality of health provider services such as low staff-to-provider ratio [46], long wait time [46], and low patient satisfaction with staff competence [31] were positively associated with LTFU. Physician’s experience in years was not associated with LTFU [40].

### Reasons for LTFU

On top of the quantitative factors presented above, 22 reasons for LTFU were qualitatively reported from six studies (Table 3) — three chronic mental [33, 36, 41] and three chronic physical [47, 50, 54] disease studies respectively. Similar to the quantitative findings reported, the reasons identified are classified into patient, clinical and healthcare provider factors.

**Table 3:** Reasons for LTFU (Qualitative findings from quantitative studies fulfilling inclusion criteria)

**Reason**	**Chronic Mental Disease**	**Chronic Physical Disease**
**Patient Factors**		
1. Away/out of town at the time of scheduled appointment		[50]
2. Belief that chronic condition was gone/not serious		[54]
3. Complicated life circumstances		[47]
4. Death	[36]	
5. Denial of diagnosis	[41]	
6. Denial of therapeutic need	[41]	
7. Felt unwell at the time of scheduled appointment		[50]
8. Financial or insurance difficulty	[33, 41]	[54]
9. Forgot about appointment		[50]
10. Moving into child's home	[36]	
11. No particular reason for default		[54]
12. No transport/clinic too far	[33, 41]	[47, 54]
13. Personal decision to discontinue care	[33, 36]	
14. Refusal to attend appointment at hospital	[36]	
15. Uncertainty of appointment dates		[47]
16. Unfamiliarity with navigating health care system		[47]
17. Work commitment		[50]
**Clinical Factors**		
18. Adverse drug events	[41]	
19. Poor functional health status	[33, 36]	
**Healthcare Provider Factors**		
20. Lack of treatment efficacy	[41]	
21. Poor patient-healthcare provider relationship	[41]	[54]
22. Transfer of care to another provider, facility, or hospice	[33, 36]	[50]

Common patient-related barriers contributing to LTFU amongst patients with chronic mental or physical diseases include transport issues [33, 41, 47, 54], and financial difficulties [33, 41, 54]. Other patient factors such as being out of town during the scheduled appointment, forgetting the appointment, uncertainty of appointment dates, work commitments, unfamiliarity with navigating the healthcare system and refusal to attend the appointment at the hospital were cited as reasons for LTFU [36, 47, 50]. Some patients also believed that their chronic conditions were not severe [54], others were in denial of their diagnoses [41] or therapeutic need [41], thus they did not attend follow-up.

For clinical factors, adverse drug effects [41] and poor functional health status [33, 36] were mentioned as reasons for LTFU. Healthcare provider factors such as the lack of treatment efficacy [41], poor patient-physician relationship [41, 54] and transferring of care to another provider or facility [33, 36, 50] were recorded as reasons contributing to LTFU.

### LTFU Definition

Terms such as ‘lost to follow-up’ [30, 33, 35, 37, 45, 47, 53], ‘dropout’ [31, 32, 34, 38, 40–42, 48, 49, 51], ‘discontinue’ [36, 43], ‘defaulters’ [52, 54], ‘attrition’ [46],’gap in care’ [44] and ‘non-attendance’ [50] were used in the included studies to refer to patients who never return for follow-up appointments or return after a clinically concerning interval. Majority of the LTFU definition included a time frame as a marker to indicate whether a patient was considered LTFU or not [31–35, 37–47, 50, 53, 54]. Given the variation of chronic diseases in the selected studies, the specified duration covers a wide spectrum, spanning from one to 24 months. However, the reason for the chosen time-period was only described in 10 studies. The duration was either selected based on clinical standards [32, 37, 39, 44, 50] or previous research studies [31, 40, 41, 46, 54]. LTFU patients were identified through clinical records [30, 32–47, 50, 52, 53] or self-reported measures [31, 43, 48, 49, 51, 54], with the former being the more common method.

## Discussion

To our knowledge, this is the first scoping review that provides a comprehensive literature review of the factors affecting LTFU among patients with chronic disease in ambulatory care of HICs. More specifically, this review analysed the factors associated with LTFU among patients with a wide range of chronic physical and/or mental diseases as opposed to considering a specific chronic disease. Overall, there was a varied spread with regards to the design, setting and sample of the studies included in this review, and most focused on patient factors with inconsistent findings. Similarly, the operational definitions of LTFU varied.

### Patient Factors

According to the studies that investigated patient factors, the association between key sociodemographic factors such as age, sex, ethnicity, education, employment type and status with LTFU were not consistent. The finding is consistent with previous systematic reviews investigating factors associated with follow-up non-attendance [17] and missed appointments [18] among patients with diabetes across countries of varying income levels. This suggests that the heterogeneous association between patient factors such as age, sex etc. with LTFU is not unique to HICs.

On the other hand, patient factors related to the accessibility of clinics such as distance from home to clinic and transportation were consistently associated with LTFU. Transport barriers [33, 41, 47, 54] and low clinic accessibility [33, 35, 45] negatively impacted follow-up. According to a recent systematic review of transport interventions and engagement in chronic care [55], interventions such as the provision of transport vouchers and chartered shuttle buses to health facilities increased healthcare utilisation among older adults with chronic illness. Therefore, decreasing transportation barriers may be an effective method in reducing LTFU. Perceived stigma, albeit a limited number of included articles investigating this factor, was a consistent factor associated with LTFU in our review [32, 43]. Stigma can come in the form of public stigma or self-stigma [56]. The impact of stigma in healthcare on patients with chronic disease is widespread [57] and plays a pivotal role in treatment engagement, especially in chronic diseases such as HIV [58] and mental illness [56, 59].

Financial factors play a crucial role in determining whether patients continue to seek healthcare treatment in HICs. Our findings revealed that for both chronic mental and physical diseases, uninsured patients [35, 48] or those who faced financial difficulty [33, 41, 54] were more prone to LTFU. Hwang et al. [60] found that chronically ill patients without insurance incurred the greatest out-of-pocket spending and were five times less prone to seek medical care. Moreover, despite subsidies or partial absorption of healthcare expenditure by the government, out-of-pocket healthcare spendings remain high among patients with chronic diseases [61]. A study reported that patients with chronic mental health diseases such as depression and anxiety incurred even higher out-of-pocket healthcare spendings [62]. Thus, it is not uncommon for patients with chronic disease to delay or forgo their treatment due to financial concerns [62, 63]. Recent evidence confirmed that health insurance coverage — public or private — increased healthcare utilisation and treatment seeking behaviour among patients with chronic conditions [64]. Therefore, it is worth investigating how to best structure the coverage of public health insurance as well as the most cost-effective and efficient way of lowering this financial barrier in different countries for patients with chronic disease(s).

Interestingly, none of the included studies investigated the association of LTFU and health literacy. According to Liu and colleagues [65], health literacy is defined as the ability of an individual to obtain and translate knowledge and information in order to maintain and improve health in a way that is appropriate to the individual and system contexts. Health literacy was found to be a strong predictor for successful self-management in patients with chronic disease [66]. This includes making appropriate healthrelated decisions such as planning for follow-up consults and treatments. Similarly, a systematic review conducted in low- and middle-income countries revealed that a lack of knowledge about the chronic disease, treatment duration and the consequences of treatment non-adherence contributed to LTFU [67]. Given the importance of health literacy, it would be worthwhile for future studies to explore if the level of health literacy as well as which aspect of health literacy is associated with LTFU in patients with chronic disease in HICs.

### Clinical factors

For clinical factors, mixed results were reported regarding the association between disease severity and LTFU. Higher disease severity may prompt patients with chronic disease to attend regular follow-up appointments to closely monitor their health conditions [68]. However, a negative correlation may be due to disease severity affecting the patient’s functional health status and hence interfere with their ability to return for follow-up appointments. This is supported by secondary qualitative findings that poor functional health status contributed to being LTFU [33, 36]. It is concerning that patients with higher disease severity may not be receiving the necessary care which can further deteriorate their health status. With the advancement of technology and the recent COVID-19 pandemic acting as a catalyst, the use of telehealth in chronic disease management has risen in popularity [69]. There is an increase in the number of studies reporting the effectiveness of telehealth in chronic disease management for chronic mental [70] and physical [71, 72] diseases. However, at this moment, it is unlikely that telehealth can fully substitute in-person care [73]. Therefore, in-person house calls can be integrated into chronic disease management especially for homebound patients or those seriously ill with chronic conditions [74]. Telehealth combined with in-person house calls may help to reduce LTFU among chronic patients with poor functional health status. More importantly, they increase the accessibility to care for this group of patients. More long-term research can explore the effectiveness of a hybrid care model using telehealth and in-person care for chronic disease management in the future.

Despite the high prevalence of patients with multiple chronic diseases in HICs [75], none of the included studies in this review investigated whether the presence of multiple chronic diseases was a factor associated with LTFU. While Wolff et al. [76] reported that having more than one chronic disease was not associated with higher non-attendance rates, another study found that patients with four or more chronic diseases were more likely to miss their follow-up appointments [77]. Given the mixed findings in existing literature, it would be beneficial to address this knowledge gap in future research. More specifically, future studies can aim to identify whether having more than one chronic disease, the number of chronic diseases as well as the type and combination of chronic diseases is associated with LTFU.

### Healthcare provider factors

This review found that lower quality of healthcare provider services is positively associated with LTFU [31, 46]. Over the years, patient-centred care (PCC) has become a paradigm for high-quality interpersonal care and is associated with decreased health care utilisation [78]. Care relationships between healthcare providers and patients play an integral role in PCC [79]. However, none of the included articles addressed interpersonal care factors. Only the qualitative findings from two studies [41, 54] attributed poor patient-healthcare provider relationship as reasons for LTFU. Better patient-physician relationship was associated with treatment and follow-up adherence among HIV patients [80]. A meta-analysis on treatment adherence also revealed that effective physician communication is significantly positively correlated with patient adherence, including appointment keeping [81]. Therefore, future interventions to reduce LTFU can focus on strategies to improve patient-physician relationship to achieve high quality PCC.

Other factors such as the healthcare provider’s years of experience was not associated with LTFU in our review. Only one study investigated this factor [40] (see supplementary material, Appendix B: Table A). Healthcare provider’s years of experience is a factor that is worth exploring as we generally assume that the greater number of years in clinical experience sharpens one’s skills and expertise, leading to better quality of patient care. However, a systematic review by Choudhry and colleagues [82] found that physicians with more experience may paradoxically be at risk for providing lower quality of care. Therefore, this is an interesting covariate to explore in future studies related to LTFU.

From the qualitative reasons collated, a crucial contributor to LTFU may be due to transfer of care [33, 36, 50]. Patients who are classified as LTFU may not be truly disengaged from care as a patient might be transferred to another care provider without informing the original care provider. This poses a challenge to the accuracy of clinics’ tracking data. According to King and colleagues [83], leveraging on health information technology advancements such as electronic health records (EHR) has greatly enhanced patient care and accessibility of patient health records. Therefore, implementing a nationwide EHR system that enables interoperability between EHR systems across healthcare providers, in the public and private sector, potentially serves a pivotal role in monitoring follow-up care among patients with chronic disease(s). If implemented successfully, this can lower the information barrier and ensures transparency in tracking a patient’s health care utilisation, reducing the risk of over or under reporting LTFU rates. Patients who become LTFU can also be easily identified for appropriate measures to be taken to re-engage them. Despite its benefits, the implementation of a nationwide EHR in HICs has progressed slower than expected, encountering multiple barriers related to users’ acceptance, management, data protection and safety [84]. Similar obstacles and high monetary costs have been cited as reasons deterring the active adoption of an EHR system in small ambulatory practice settings [85]. Nevertheless, a centralised database with patients’ health and medical records is a crucial tool for monitoring follow-up appointments. Future research can expand on Fragidis and Chatzoglou’s findings [84], to explore targeted ways to successfully execute nationwide EHR in different countries with differing health systems characteristics.

### Operational definition of LTFU

Based on the included studies, the operational definition of LTFU varied drastically. For instance, even for the same chronic disease such as diabetes, the time-period chosen to ascertain LTFU patients differed across studies. Simmons et al. [54] selected a 10-month interval while Masuda et al. [38] used a 12-month period of no clinic contact. The former study set this interval based on prior research evidence [54] whereas the latter study did not specify any reason for selecting a 12-month interval [38]. These differences decrease the comparability across studies, making it challenging to study LTFU even for the same chronic disease. Furthermore, a diverse range of terms were used to represent LTFU in the included articles. Many other terms such as ‘lapse in care’ and ‘prolonged gap in care’ [24] were used as synonyms of LTFU in existing literature. Attempts to standardise the operational definition of LTFU for specific chronic diseases as well as the terms used to represent LTFU would be valuable and enhance comparability of future study findings.

## Limitations

Our scoping review has several limitations. We excluded grey literature and did not conduct reference chaining. While this may imply that some relevant articles may not have been included, the use of a comprehensive search strategy suggests that most of the relevant studies would have been included. We also only included studies that were published in the English language. As our review was interested in overall LTFU from ambulatory care, we excluded studies related to dropping out of specific treatment modalities (i.e., not usual standard clinical care) or treatment programmes. Due to the specificity of these studies, their results may not be generalisable to the broader scope of LTFU in ambulatory care. It is also important to note that although the list of HICs is unlikely to change drastically, the list is updated yearly and this review’s HICs were determined based on the World Bank country classification published in 2021 [27]. Therefore, by the time this review is published, the list of HICs might be slightly different. Additionally, this review aimed to look at factors that were significantly associated with LTFU after controlling for covariates. As a result, this strict criterion excluded 11 studies that did not control for confounders (Fig. 1). Confounding factors may affect the association between the dependent and independent variable through masking a true association or falsely demonstrating an apparent association [86]. Hence, the exclusion of articles which did not adjust for confounders reduces bias, improving the study’s credibility. Finally, patients identified as LTFU may not be truly disengaged from ambulatory care because the patient might be transferred to another healthcare system without notifying the original healthcare provider.

## Conclusion

This scoping review identified 32 factors associated with LTFU among patients with chronic disease in the ambulatory setting of HICs. The directionality and association across studies are largely inconsistent. Nevertheless, financial factors (i.e., no insurance coverage) and low accessibility of care in terms of travel distance to clinic are factors that were significantly positively associated with LTFU. We also found that the operational definitions and terms used to represent LTFU varied greatly across studies. Our findings highlight the importance of considering patient, clinical and healthcare provider factors associated with LTFU when planning appropriate policies or interventions in reducing LTFU. This review also highlights the importance of adjusting for potential confounders of LTFU. Future research should explore the relationship between stigma and LTFU as well as how interpersonal care factors influence follow-up behaviour to better understand the root cause of patients discontinuing follow-up appointments. Further research using a qualitative methodological approach to understand the reasons contributing to LTFU will be useful and a more direct method to develop targeted strategies to increase follow-up engagement. Given the heterogeneity of the operational definition of LTFU used in various studies, further work on reducing LTFU would need to standardise the definition and ways of measuring it at the first instance.

### Supplementary Information

**Additional file 1: Appendix A.** Final Search Strategy and Search Results. **Appendix B Table A.** Significant factors associated with LTFU (detailed version with directionality).

## Data Availability

All data generated or analysed during this study are included in its published article and its supplementary information files.

## Abbreviations

BMI: Body Mass Index
EHR: Electronic Health Records
HIC: High-income countries
LMIC: Low- and middle-income countries
HIV: Human Immunodeficiency Virus
LTFU: Lost to follow-up
PCC: Patient Centred Care
