# Supplementary material for: A scoping review on the factors associated with the lost to follow-up (LTFU) amongst patients with chronic disease in ambulatory care of high-income countries (HIC)
Source: BMC Health Serv Res. 2023 Aug 22;23:883. doi: 10.1186/s12913-023-09863-0 (PMC10464417; doi:10.1186/s12913-023-09863-0)
Supplement: Supplementary file 1 — Additional file 1: Appendix A. Final Search Strategy and Search Results. Appendix B Table A. Significant factors associated with LTFU (detailed version with directionality). [file 12913_2023_9863_MOESM1_ESM.docx]

**Appendix A**

Final Search Strategy and Search Results

Date of search: 07/01/2022

# Medline (Ovid)

| Concepts |  | Search Terms | Results |
| --- | --- | --- | --- |
| **Chronic Disease(s)** | 1 | chronic disease/ or Noncommunicable Diseases/ or exp Diabetes Mellitus/ or exp Hypertension/ or exp Hyperlipidemias/ or exp Stroke/ or exp Asthma/ or exp Pulmonary Disease, Chronic Obstructive/ or exp Schizophrenia/ or exp Depressive Disorder, Major/ or exp Bipolar Disorder/ or exp Dementia/ or exp Osteoarthritis/ or exp Prostatic Hyperplasia/ or exp Anxiety Disorders/ or exp Parkinson Disease/ or exp Renal Insufficiency, Chronic/ or exp Epilepsy/ or exp Osteoporosis/ or exp psoriasis/ or exp Arthritis, Rheumatoid/ or exp Myocardial Ischemia/ | 2740231 |
|  | 2 | ((Chronic* adj (disease* or illn* or ill or condition*)) or (Chronic disease* adj (non-communicable or noncommunicable or non communicable)) or (Disease* adj (non-communicable or noncommunicable or non communicable or non-infectious or noninfectious or non infectious))).ab,ti,kw. | 111240 |
|  | 3 | (Diabetes mellitus or pre-diabetes or Hypertension or Hyperlipidemia or Stroke or Asthma or Chronic obstructive pulmonary disease or Schizophrenia or Major depression or Bipolar disorder or Dementia or Osteoarthritis or Benign prostatic hyperplasia or Anxiety disorder or Parkinson's disease or chronic kidney disease or nephrosis or nephritis or Epilepsy or Osteoporosis or Psoriasis or Rheumatoid arthritis or ((Ischemic or ischaemic) adj heart disease)).ab,ti,kw. | 1927455 |
|  | 4 | 1 or 2 or 3 | 3506904 |
| **Lost to follow-up** | 5 | Patient Dropouts/ or "Lost to Follow-Up"/ | 9706 |
|  | 6 | (((lost to or loss to or default) adj follow*) or Default* or ((Treatment or care or healthcare) adj2 (default* or dropout)) or (dropout* adj patient*) or dropout* or attrition).ab,ti,kw. | 52238 |
|  | 7 | 5 or 6 | 59129 |
| **Ambulatory care** | 8 | Ambulatory Care/ or General Practice/ or family practice/ | 121270 |
|  | 9 | ((Ambulatory adj2 Care) or ((Outpatient or specialist) adj2 (Care or Service* or Clinic*)) or clinic or outpatient or (General adj Practice) or (Family adj Practice*)).ab,ti,kw. | 441925 |
|  | 10 | 8 or 9 | 512357 |
|  | 11 | 4 and 7 and 10 | 808 |
|  | 12 | Limit 11 to English language | 773 |

# Web of Science

| Concepts |  | Search Terms | Results |
| --- | --- | --- | --- |
| **Chronic Disease(s)** | 1 | TS=((Chronic* NEAR/0 (disease* or illn* or ill or condition*)) or (Chronic disease* NEAR/0 (non-communicable or noncommunicable or "non communicable")) or (Disease* NEAR/0 (non-communicable or noncommunicable or "non communicable" or non-infectious or noninfectious or "non infectious"))) | 142743 |
|  | 2 | TS=(Diabetes mellitus or pre-diabetes or Hypertension or Hyperlipidemia or Stroke or Asthma or Chronic obstructive pulmonary disease or Schizophrenia or Major depression or Bipolar disorder or Dementia or Osteoarthritis or Benign prostatic hyperplasia or Anxiety disorder or Parkinson's disease or chronic kidney disease or nephrosis or nephritis or Epilepsy or Osteoporosis or Psoriasis or Rheumatoid arthritis or ((Ischemic or ischaemic) NEAR/0 heart disease)) | 2807925 |
|  | 3 | #1 or #2 | 2906051 |
| **Lost to follow-up** | 4 | TS=((("lost to" or "loss to" or default) NEAR/0 follow*) or Default* or ((Treatment or care or healthcare) NEAR/2 (default* or dropout)) or (dropout* NEAR/0 patient*) or dropout* or attrition) | 132108 |
| **Ambulatory care** | 5 | TS=((Ambulatory NEAR/2 Care) or ((Outpatient or specialist) NEAR/2 (Care or Service* or Clinic*)) or clinic or outpatient or (General NEAR/0 Practice) or (Family NEAR/0 Practice*)) | 553284 |
|  | 6 | #3 and #4 and #5 | 1216 |
|  | 7 | #3 AND #4 AND #5 and English (Languages) | 1200 |

# CINAHL

| Concepts |  | Search Terms | Results |
| --- | --- | --- | --- |
| **Chronic Disease(s)** | 1 | (MH "Chronic Disease+") OR (MH "Diabetes Mellitus+") OR (MH "Hypertension+") OR (MH "Hyperlipidemia+") OR (MH "Stroke+") OR (MH "Asthma+") OR (MH "Pulmonary Disease, Chronic Obstructive+") OR (MH "Schizophrenia+") OR (MH "Depression+") OR (MH "Bipolar Disorder+") OR (MH "Dementia+") OR (MH "Osteoarthritis+") OR (MH "Prostatic Hypertrophy+") OR (MH "Anxiety Disorders+") OR (MH "Parkinson Disease+") OR (MH "Renal Insufficiency, Chronic+") OR (MH "Epilepsy+") OR (MH "Osteoporosis") OR (MH "Psoriasis+") OR (MH "Arthritis, Rheumatoid+") OR (MH "Myocardial Ischemia+") | 944,734 |
|  | 2 | TI( (Chronic* N0 (disease* or illn* or ill or condition*)) or (Chronic disease* N0 (non-communicable or noncommunicable or non communicable)) or (Disease* N0 (non-communicable or noncommunicable or non communicable or non-infectious or noninfectious or non infectious)) )  OR  AB ( (Chronic* N0 (disease* or illn* or ill or condition*)) or (Chronic disease* N0 (non-communicable or noncommunicable or non communicable)) or (Disease* N0 (non-communicable or noncommunicable or non communicable or non-infectious or noninfectious or non infectious)) ) | 58,212 |
|  | 3 | TI ( Diabetes mellitus or pre-diabetes or Hypertension or Hyperlipidemia or Stroke or Asthma or Chronic obstructive pulmonary disease or Schizophrenia or Major depression or Bipolar disorder or Dementia or Osteoarthritis or Benign prostatic hyperplasia or Anxiety disorder or Parkinson's disease or chronic kidney disease or nephrosis or nephritis or Epilepsy or Osteoporosis or Psoriasis or Rheumatoid arthritis or ((Ischemic or ischaemic) adj heart disease) )  OR  AB ( Diabetes mellitus or pre-diabetes or Hypertension or Hyperlipidemia or Stroke or Asthma or Chronic obstructive pulmonary disease or Schizophrenia or Major depression or Bipolar disorder or Dementia or Osteoarthritis or Benign prostatic hyperplasia or Anxiety disorder or Parkinson's disease or chronic kidney disease or nephrosis or nephritis or Epilepsy or Osteoporosis or Psoriasis or Rheumatoid arthritis or ((Ischemic or ischaemic) adj heart disease) ) | 534,513 |
|  | 4 | 1 or 2 or 3 | 1,130,115 |
| **Lost to follow-up** | 5 | (MH "Patient Dropouts") | 2,238 |
|  | 6 | TI ( ((lost to or loss to or default) N0 follow*) or Default* or ((Treatment or care or healthcare) N2 (default* or dropout)) or (dropout* N0 patient*) or dropout* or attrition )  OR  AB ( ((lost to or loss to or default) N0 follow*) or Default* or ((Treatment or care or healthcare) N2 (default* or dropout)) or (dropout* N0 patient*) or dropout* or attrition ) | 20,979 |
|  | 7 | 5 or 6 | 22,351 |
| **Ambulatory care** | 8 | (MH "Ambulatory Care") OR (MH "Family Practice") OR (MH "Outpatient Service") | 49,162 |
|  | 9 | TI ( (Ambulatory N2 Care) or ((Outpatient or specialist) N2 (Care or Service* or Clinic*)) or clinic or outpatient or (General N0 Practice) or (Family N0 Practice*) )  OR  AB ( (Ambulatory N2 Care) or ((Outpatient or specialist) N2 (Care or Service* or Clinic*)) or clinic or outpatient or (General N0 Practice) or (Family N0 Practice*) ) | 209,048 |
|  | 10 | 8 or 9 | 234,489 |
|  | 11 | 4 and 7 and 10 | 538 |
|  | 12 | Limit 11 to English language | 534 |

# Embase

| Concepts |  | Search Terms | Results |
| --- | --- | --- | --- |
| **Chronic Disease(s)** | 1 | 'chronic disease'/de OR 'non communicable disease'/de OR 'diabetic complication'/exp OR 'hypertension'/exp OR 'hyperlipidemia'/exp OR 'cerebrovascular accident'/exp OR 'asthma'/exp OR 'chronic obstructive lung disease'/exp OR 'schizophrenia'/exp OR 'major depression'/exp OR 'bipolar disorder'/exp OR 'dementia'/exp OR 'osteoarthritis'/exp OR 'prostate hypertrophy'/exp OR 'anxiety disorder'/exp OR 'Parkinson disease'/de OR 'chronic kidney failure'/exp OR 'epilepsy'/exp OR 'osteoporosis'/exp OR 'psoriasis'/exp OR 'rheumatoid arthritis'/exp OR 'ischemic heart disease'/exp | 4,404,007 |
|  | 2 | ((chronic* NEAR/1 (disease* OR illn* OR ill OR condition*)):ti,ab,kw) OR (('chronic disease*' NEAR/1 ('non communicable' OR noncommunicable OR 'non communicable')):ti,ab,kw) OR ((disease* NEAR/1 ('non communicable' OR noncommunicable OR 'non communicable' OR 'non infectious' OR noninfectious OR 'non infectious')):ti,ab,kw) | 183,577 |
|  | 3 | 'diabetes mellitus':ti,ab,kw OR 'pre diabetes':ti,ab,kw OR hypertension:ti,ab,kw OR hyperlipidemia:ti,ab,kw OR stroke:ti,ab,kw OR asthma:ti,ab,kw OR 'chronic obstructive pulmonary disease':ti,ab,kw OR schizophrenia:ti,ab,kw OR 'major depression':ti,ab,kw OR 'bipolar disorder':ti,ab,kw OR dementia:ti,ab,kw OR osteoarthritis:ti,ab,kw OR 'benign prostatic hyperplasia':ti,ab,kw OR 'anxiety disorder':ti,ab,kw OR 'parkinson disease':ti,ab,kw OR 'chronic kidney disease':ti,ab,kw OR nephrosis:ti,ab,kw OR nephritis:ti,ab,kw OR epilepsy:ti,ab,kw OR osteoporosis:ti,ab,kw OR psoriasis:ti,ab,kw OR 'rheumatoid arthritis':ti,ab,kw OR (((ischemic OR ischaemic) NEAR/1 'heart disease'):ti,ab,kw) | 2,826,899 |
|  | 4 | #1 or #2 or #3 | 5,205,836 |
| **Lost to follow-up** | 5 | 'patient dropout'/de | 2,032 |
|  | 6 | ((('lost to' OR 'loss to' OR default) NEAR/1 follow*):ti,ab,kw) OR default*:ti,ab,kw OR (((treatment OR care OR healthcare) NEAR/3 (default* OR dropout)):ti,ab,kw) OR ((dropout* NEAR/1 patient*):ti,ab,kw) OR dropout*:ti,ab,kw OR attrition:ti,ab,kw | 113,343 |
|  | 7 | #5 or #6 | 114,046 |
| **Ambulatory care** | 8 | 'ambulatory care'/de OR 'general practice'/de OR 'outpatient care'/de | 167,980 |
|  | 9 | ((ambulatory NEAR/3 care):ti,ab,kw) OR (((outpatient OR specialist) NEAR/3 (care OR service* OR clinic*)):ti,ab,kw) OR clinic:ti,ab,kw OR outpatient:ti,ab,kw OR ((general NEAR/1 practice):ti,ab,kw) OR ((family NEAR/1 practice*):ti,ab,kw) | 749,009 |
|  | 10 | #8 or #9 | 832,521 |
|  | 11 | #4 and #7 and #10 | 2,413 |
|  | 12 | #11 AND [english]/lim | 2,343 |

# PsycINFO (Ovid)

| Concepts |  | Search Terms | Results |
| --- | --- | --- | --- |
| **Chronic Disease(s)** | 1 | exp chronic illness/ or exp Diabetes Mellitus/ or exp Hypertension/ or exp Lipid Metabolism Disorders/ or exp Cerebrovascular Accidents/ or exp Asthma/ or exp Chronic Obstructive Pulmonary Disease/ or exp Schizophrenia/ or exp major depression/ or exp bipolar disorder/ or exp Dementia/ or exp Arthritis/ or exp anxiety disorder/ or exp Parkinson's Disease/ or exp kidney diseases/ or exp Epilepsy/ or exp Osteoporosis/ or exp skin disorders/ or exp heart disorders/ | 517886 |
|  | 2 | ((Chronic* adj (disease* or illn* or ill or condition*)) or (Chronic disease* adj (non-communicable or noncommunicable or non communicable)) or (Disease* adj (non-communicable or noncommunicable or non communicable or non-infectious or noninfectious or non infectious))).ab,ti,id. | 29706 |
|  | 3 | (Diabetes mellitus or pre-diabetes or Hypertension or Hyperlipidemia or Stroke or Asthma or Chronic obstructive pulmonary disease or Schizophrenia or Major depression or Bipolar disorder or Dementia or Osteoarthritis or Benign prostatic hyperplasia or Anxiety disorder or Parkinson's disease or chronic kidney disease or nephrosis or nephritis or Epilepsy or Osteoporosis or Psoriasis or Rheumatoid arthritis or ((Ischemic or ischaemic) adj heart disease)).ab,ti,id. | 369120 |
|  | 4 | 1 or 2 or 3 | 615501 |
| **Lost to follow-up** | 5 | treatment dropouts/ | 2676 |
|  | 6 | (((lost to or loss to or default) adj follow*) or Default* or ((Treatment or care or healthcare) adj2 (default* or dropout)) or (dropout* adj patient*) or dropout* or attrition).ab,ti,id. | 28797 |
|  | 7 | 5 or 6 | 29756 |
| **Ambulatory care** | 8 | outpatient treatment/ or family medicine/ | 8537 |
|  | 9 | ((Ambulatory adj2 Care) or ((Outpatient or specialist) adj2 (Care or Service* or Clinic*)) or clinic or outpatient or (General adj Practice) or (Family adj Practice*)).ab,ti,id. | 93919 |
|  | 10 | 8 or 9 | 96028 |
|  | 11 | 4 and 7 and 10 | 274 |
|  | 12 | Limit 11 to English language | 257 |

**Appendix** **B**

Table A: Significant factors associated with LTFU (detailed version with directionality)

| **Study/**  **Country** | **Statistics** | **Covariates** | **Patient Factors** | **Clinical Factors** | **Healthcare Provider Factors** |
| --- | --- | --- | --- | --- | --- |
| **Chronic Mental Disease** | | | | | |
| Berghofer et al. (2002)/ Austria | Multivariate logistics regression | Age, Gender/Sex, Marital status, Education level, Patient expectations, Patient self-rating of disease severity (Clinical Global Impression), Clinician-rated disease severity (Clinical Global Impression), Clinician-rated global functioning, Global Assessment of Functioning), Referral type, Number of overall supporters (Social Support Questionnaire), Quality of life with work (Berlin Quality of Life Profile) | (+) Unemployment, (-) Availability of home care, (-) Living alone, (-) High quality of patient's living situation (Berlin Quality of Life Profile), (-) High quality of patient's relationship with family/significant others (Berlin Quality of Life Profile) | (+) Received previous psychiatric treatment, (+) High self-rated Global Assessment of Functioning, (-) Schizophrenia diagnosis | (+) Low patient statisfaction with staff competance |
| Bowersox et al. (2013)/ USA | Logistic regression analysis | Ethnicity/Race, Global Assessment of Functioning score at admission, Inpatient days in year prior, Number of lifetime inpatient admissions, Diagnostic status, Expected social stigma from treatment, Expected material cost of treatment, Expected social consequences of treatment, Expected symptom change from treatment, Perceived treatment support from family, Perceived treatment support from treatment staff, Psychiatrist appointment attendance | (+) Younger age, (+) Male, (+) Higher expectations of experiencing internal stigma as a result of treatment, (+) Less frequent medication pick up from pharmacy |  | (+) Less short-term attendance at group therapy |
| Boyd et al. (2022)/ USA | Multivariate model | Age, Ethnicity/Race, Preferred language, Clinical dementia diagnosis | (+) Female, (+) Lower education level, (+) Dying in long-term care facility, (+) Greater distance between home and clinic |  |  |
| Ezquiaga et al. (2014)/ Spain | Multivariate logistic regression analysis | Age, Current drug abuse/dependence, Current alcohol abuse/dependence, Presence of Axis I comorbidity, Years with illness, Years of diagnosis delay | (+) Current smoker, (+) History of poor treatment compliance, (+) History of medium treatment compliance | (+) Seasonality (yes) |  |
| Fernandez et al. (2021)/ Multiple countries* | Predictors of dropout were examined with survival analysis using a logistic link function | **Dropout after 1-2 visits** Age, Gender/Sex, Education level, Marital status, Employed, Family income level, Health insurance, Disorder type, Type of treatment/care |  | (+) Mild severity of disorder, (+) Moderate severity of disorder |  |
|  |  | **Dropout after 3+ visits** Age, Gender/Sex, Education level, Marital status, Employed, Family income level, Severity of disorder, Disorder type, Type of treatment/care | (+) No health insurance |  |  |
| Hishikawa et al. (2017)/ Japan | Multiple logistic regression analysis | Cognitive function measurements:  Mini-Mental State Examination, Hasegawa Dementia Score-Revised, Frontal Assessment Battery, Montreal Cognitive Assessment , Geriatric Depression Scale , Apathy Scale |  | (+) Low score on Abe's behvaioural and psychological symptoms of dementia (Deterioration of behavioural and psychological symptoms of dementia for University Hospital patients), (+) Activities of daily living decline (for Total patients) |  |
| Lerner et al. (2012)/ Israel | Logistic regression analysis | Age, Gender/Sex, Presence of chronic physical conditions, Severity of any mood or anxiety disorder, Treatment with psychotropic drugs |  |  | (+) Treatment under general medical sector |
| Minamisawa et al. (2016)/ Japan | Multivariate cox regression analyses | Gender/Sex, Occupation, Diagnosis type, Severity of illness (Clinical Global Impression), Presence of previous treatment, Psychiatrist's gender, Psychiatrists's marital status, Psychiatrist's experience in years | (+) Younger age (anxiety disorder patients), (+) Low education level (mood disorder patients), (+) Divorced/widowed (mood disorder patients) | (-) GAF score of 51-60 (anxiety disorder patients) | (-) Patient-physician sex concordance (anxiety disorder patients) |
| Moon et al. (2012)/ South Korea | Cox proportional hazard regression model | Age, Gender/Sex, Bipolar subtype, Mood state at time of first visit | (+) Presence of past history of dropout | (-) Previous diagnosis of bipolar disorder, (-) Previous diagnosis of other axis I disorder, (-) Presence of past psychotic symptoms, (-) Longer duration of bipolar disorder |  |
| Simon et al. (2010)/ USA | Logistic regression | Age, Healthcare provider type, Self-rated importance of initiating psychotherapy, Interaction between severity of depression score and provider type | (-) Male | (-) High Patient Health Questionnaire scores (severe depression symptoms) |  |
| Sirey et al. (2001)/ USA | Logistic regression | Age, Perceived stigma (Stigma coping scale), Depression severity (Hamilton Depression scale) | (+) Interaction effect of perceived stigma by age ≥65 (In older patients, greater perceived stigma positively associated with LTFU) |  |  |
| **Study/**  **Country** | **Statistics** | **Covariates** | **Patient Factors** | **Clinical Factors** | **Healthcare Provider Factors** |
| **Chronic Physical Disease** | | | | | |
| Ballard et al. (1988)/ USA | Multiple Logistic regression analysis | Age, Diastolic blood pressure, Continuity of care |  | (+) Obese weight | (+) Physicans with lower drug aggressiveness, (+) Less intense prior contact with medical care system |
| Buys et al. (2019)/ USA | Binary logistics regression | Age, Gender/Sex, Ethnicity/Race, Total number of appointments | Insignificant findings |  |  |
| Chow et al. (2011)/ Hong Kong SAR, China | Multivariate logistic analysis | Patients with a living spouse | (+) Younger age |  |  |
| Gao et al. (2019)/ USA | Multivariate analysis | Gender/Sex, Regional average adjusted gross income |  | (+) Baseline visual acuity of 20/50-20/100, (+) Baseline visual acuity <20/100, (-) Presence of Branch retinal vein occlusion |  |
| Khanh et al. (2020)/ USA | Multivariable analysis | **LTFU 1 month** | (+) Black, (+) Hispanic, (+) Other race, (+) Unknown race, (+) Greater distance between home and clinic, (+) No health insurance coverage, (-) Older age (specifically 65-80) |  | (-) Seeing another non-vascular surgeon specialist at Northwestern Memorial Hospital |
|  |  | **LTFU 1 Year** Number of rescheduled post-op appointments |  | (-) Undergone reintervention (complication during hospitalisation) | (-) Seeing another non-vascular surgeon specialist at Northwestern Memorial Hospital |
| Kim et al. (2021)/ South Korea | Multivariate regression analysis |  | (+) Male | (+) Diabetes mellitus (DM)duration ≤5 years, (+) Presence of persistent diabetic macular edema (DME) |  |
| Masuda et al. (2006)/ Japan | Unconditional logistic regression | Age, Gender/Sex, Health complications (retinopathy/ renal dysfunction), Previous DM diagnosis |  | (-) Prescription of insulin injection medication, (-) Prescription of oral agents medication |  |
| Mathieu et al. (2014)/ USA | Logistic regression model | Age, Ethnicity/Race, Income, History of gestational diabetes mellitus / delivery of a large-for-gestational-age baby, Gestational age at diagnosis, Type of treatment received (diet/exercise; glyburide; insulin), Infant birth weight | (+) Lower education level | (+) Higher BMI at diagnosis, (+) Higher fasting blood glucose level |  |
| Shiu et al. (2019)/ Hong Kong SAR, China | Unconditional logistics regression | Age, Education level, Duration of diabetes, Usage of anti-diabetic medication, Length of appointment interval | (-) Female | (+) Higher HbA1c level, (+) Presence of diabetic retinopathy (DR) |  |
| Simmons et al. (2007)/ New Zealand | Logistic regression | Age, Ethnicity/Race, Income, Knowledge level, Tertiary education, Current smoker, Community service card, Employed, Diastolic blood pressure, Systolic blood pressure, How Type 2 diabetes was found, HbA1c level, Duration of diabetes, Nail/skin care |  | (+) Presence of callus, (-) Presence of severe/proliferative retinopathy, (-) Prescription of insulin therapy, (-) Prescription of blood pressure medication, (-) Prescription of antihyperglycaemic medication |  |
| Sonoda et al. (2020)/ Japan | Multivariable logistic regression model | Hours of housework, childcare and care, Duration of diabetes | (-) Older age, (-) Higher supervisor support (work-related factor) | (-) Presence of metabolic syndrome |  |
| Szadkowski et al. (2018)/ Canada | Multivariable Generalised Estimating Equations (GEE) | Gender and men having sex with men, CD4 cell count | (+) Younger age, (+) White race, (+) Injection drug use | (+) Longer duration of Human Immunodeficiency Virus, (-) Viral load <50 copies/mL | (+) Earlier calender year of inter-visit interval |
| Tsui et al. (2016)/ USA | Logistics regression | Presence of diabetic retinopathy, HbA1c level | (+) Greater distance between home and clinic |  |  |
| **Study/**  **Country** | **Statistics** | **Covariates** | **Patient Factors** | **Clinical Factors** | **Healthcare Provider Factors** |
| **Unspecified Chronic Disease** | | | | | |
| Yoon et al. (2020)/ USA | Multilevel logistic regression | Age, Gender/Sex, Ethnicity/Race, Marital status, Service-connected diasbility, Means test category, Rurality, Baseline healthcare costs, Gagne comorbidity score, Presence of mental health condition, Practice size, Area-level (clinic location) median household income, Distance between home and nearest Veterans Health Administration clinic |  |  | (+) Low Patient Aligned Care Team implementation score, (+) Veterans Health Administration-medical center-based clinic, (+) Low staff-to-provider ratio, (+) Long wait time for new patients' appointment, (-) High mean score in the respective Patient Aligned Care Team implementation domain: Access, Care coordination, Comprehensiveness, Self-management support, Communications, Shared decision-making |
| *Multiple countries included: Argentina (not classified as High-income Country in the 2021 World Bank List); Belgium; France; Germany; Israel; Italy; Japan; Netherlands; New Zealand; Northern Ireland; Poland; Portugal; Spain; United States | | | | | |
